# Curcumin as Treatment for Bladder Cancer: A Preclinical Study of Cyclodextrin-Curcumin Complex and BCG as Intravesical Treatment in an Orthotopic Bladder Cancer Rat Model

**DOI:** 10.1155/2018/9634902

**Published:** 2018-06-10

**Authors:** J. Falke, J. Parkkinen, L. Vaahtera, C. A. Hulsbergen-van de Kaa, E. Oosterwijk, J. A. Witjes

**Affiliations:** ^1^Radboud University Nijmegen Medical Centre, Department of Urology, Nijmegen, Netherlands; ^2^Institute of Biomedicine, University of Helsinki, Department of Biochemistry and Developmental Biology, Helsinki, Finland; ^3^Radboud University Nijmegen Medical Centre, Department of Pathology, Nijmegen, Netherlands

## Abstract

**Objective:**

To evaluate the antitumor effect of cyclodextrin-curcumin complex (CDC) on human and rat urothelial carcinoma cells* in vitro* and to evaluate the effect of intravesical instillations of CDC, BCG, and the combination* in vivo* in the AY-F344 orthotopic bladder cancer rat model. Curcumin has anticarcinogenic activity on urothelial carcinoma and is therefore under investigation for the treatment of non-muscle invasive bladder cancer. Curcumin and BCG share immunomodulating pathways against urothelial carcinoma.

**Methods:**

Curcumin was complexed with cyclodextrin to improve solubility. Four human urothelial carcinoma cell lines and the AY-27 rat cell line were exposed to various concentrations of CDC* in vitro*. For the* in vivo* experiment, the AY-27 orthotopic bladder cancer F344 rat model was used. Rats were treated with consecutive intravesical instillations of CDC, BCG, the combination of CDC+BCG, or NaCl as control.

**Results:**

CDC showed a dose-dependent antiproliferative effect on all human urothelial carcinoma cell lines tested and the rat AY-27 urothelial carcinoma cell line. Moreover, intravesical treatment with CDC and CDC+BCG results in a lower percentage of tumors (60% and 68%, respectively) compared to BCG (75%) or control (85%). This difference with placebo was not statistically significant (p=0.078 and 0.199, respectively). However, tumors present in the placebo and BCG-treated rats were generally of higher stage.

**Conclusions:**

Cyclodextrin-curcumin complex showed an antiproliferative effect on human and rat urothelial carcinoma cell lines* in vitro*. In the aggressive orthotopic bladder cancer rat model, we observed a promising effect of CDC treatment and CDC in combination with BCG.

## 1. Introduction

New treatment options for patients with non-muscle invasive bladder cancer (NMIBC) are urgently needed. Despite transurethral resection and adjuvant treatment with intravesical instillations, these bladder tumors recur in around one-third to two-thirds of patients, depending on risk category [1, 2]. Based on tumor characteristics and other parameters, the NMIBC-patient is classified in a risk category and treated adjuvantly: in general, intermediate-risk patients receive intravesical chemotherapy whereas high-risk patients are treated with intravesical Bacillus Calmette Guérin (BCG) [2]. Even after BCG treatment for 1-3 years, the 5-yr risk of recurrence is 41.3% [1].

Besides suboptimal oncological results, there are also considerable side effects ranging from mild urogenital complaints to severe systemic side effects. Additionally, due to recent BCG shortage, future alternatives for adjuvant treatment are more than welcome.

Curcumin is a component of dried turmeric powder, a food spice derived from the plant* Curcuma Longa*. Curcumin is a nontoxic agent, with anti-inflammatory, antioxidant, and anticarcinogenic activity [3, 4], including apoptotic effects on urothelial carcinoma (UC)* in vitro *[5, 6] and* in vivo *[7–10]. The molecular mechanisms underlying the antitumor effect have not been fully unraveled. Treatment with curcumin leads to downregulation of cell-signaling pathways responsible for proliferation, invasion, and angiogenesis [7, 11–13] by suppressing activating transcription factors like NF-*κ*B [7, 14].

Curcumin also enhances pathways mediated by tumor necrosis factor-related apoptosis-inducing ligand (TRAIL), an apoptosis-inducing cytokine that targets human cancer cells specifically [15]. Interestingly, induction and expression of TRIAL by polymorphonuclear neutrophils are a key step in the therapeutic effect of BCG treatment for NMIBC [13]: the relative specificity of TRAIL to tumor cells may explain the selectivity of the BCG-induced immune response against bladder cancer [13]. Curcumin may potentiate the efficacy of BCG, since it upregulates the expression of DR-5, one of the main receptors for TRAIL [7, 15]. Sharing pathways responsible for tumor selective apoptosis, curcumin, and BCG may have a synergistic antiproliferative effect on UC [7].

Because the aqueous solubility of curcumin is low, the bioavailability after systemic or topical administration is modest [16]. Complexing the curcumin to cyclodextrin improves the aqueous solubility [17], thereby increasing its cellular uptake and efficacy* in vitro* and* in vivo *[18, 19]. Using a pH shift method, we prepared a concentrated aqueous solution of cyclodextrin-curcumin complex (CDC) which is stable even in the presence of only two molar excess of cyclodextrin (L. Vaahtera and J. Parkkinen et al., unpublished results), suggestive of improved bioavailability.

In view of the antitumor effects of curcumin on UC, the accessibility of the bladder for local therapy, and the potential synergistic effect of CDC and BCG, we hypothesized that CDC may be a treatment modality for NMIBC. In this preclinical study, we tested the antiproliferative effect of CDC on various bladder cancer cell lines* in vitro* and the efficacy of CDC, BCG, and a combination of CDC+BCG as intravesical treatment for bladder cancer in a syngeneic orthotopic bladder cancer model in rats.

## 2. Materials and Methods

### 2.1. Study Drugs

Cyclodextrin-curcumin (CDC) 12 mg/ml was prepared by a pH shift method: In a 0.18 mol/L sodium hydroxide solution, 112 g/L hydroxypropyl-*γ*-cyclodextrin (Wacker Chemie, Munich Germany) and 15 g/L curcumin (Sabinsa Corporation, East Windsor, NJ, USA) were dissolved and the pH was adjusted to 6.0 using hydrochloric acid and citric acid. This method yields a concentrated aqueous solution of curcumin (32 mmol/l) in the presence of 2-fold molar excess of hydroxypropyl-*γ*-cyclodextrin. The stock solution was sterile filtered and diluted to the proper concentrations with serum-free RPMI-1640 medium (Invitrogen, Carlsbad, California, USA) for* in vitro* experiments or with 0.9% NaCl for* in vivo* experiments. This CDC solution meets the pharmaceutical requirements for intravascular administration.

For the* in vivo* experiments, BCG-Medac (Medac GmbH, Hamburg, Germany), RIVM strain 1173-P2, 2×10^8^ – 3×10^9^ colony forming units were used. It was resuspended in 0.9% NaCl within 10 minutes before use.

### 2.2. In Vitro Experiment

#### 2.2.1. Cell Lines and Conditions

Human UC cell lines RT4, RT112, 253J, and T24 represent well-differentiated, moderately differentiated (2x), and poorly differentiated phenotypes of UC, respectively. The AY-27 rat UC cell line used was established from a primary bladder tumor in FANFT (N-[4-(5-nitro-2-furyl)-2-thiazolyl]formamide) fed Fischer F344 rats. Cells were cultured as a monolayer in RPMI-1640 medium with L-glutamine (Invitrogen, Carlsbad, California, USA), supplemented with 10% fetal calf serum (Sigma-Aldrich, St. Louis, Missouri, USA), 100 U/mL penicillin G, and 100 *μ*g/mL streptomycin (Invitrogen, Carlsbad, California, USA) in a humidified 95% air/5% CO_2_ atmosphere at 37°C. The medium was replaced twice a week. When confluent, cells were split using standard trypsinisation procedures.

#### 2.2.2. Cell Viability

Cells were seeded in 96-well plates at 1*∗*10^4^ cells/well in 100*μ*L culture medium. After 24 hours, the medium was replaced by 100*μ*L study drug. CDC was dissolved in serum-free medium at 0.6125 to 640 *μ*mol/L. The AY27 cells were treated for 0.5, 1, or 2 hours and the human cell lines for 1 hour. After treatment, the cells were rinsed three times using serum-free medium, then 100*μ*L normal medium was added. At one or three days, a MTT cell viability assay was performed according to the manufacturers' instruction (Sigma-Aldrich Co, St. Louis, Missouri, USA). The experiments were performed in quadruplicate.

### 2.3. In Vivo Experiment

#### 2.3.1. Animals

Study design and animal procedures were approved by the Institutional Animal Care and Use Committee (IACUC), Committee for Animal Experiments (Radboud University Nijmegen Medical Centre, The Netherlands), and in compliance with Dutch and European regulations. Eighty Fischer F344 rats were purchased (Charles River, L'Arbresle Cedex, France) and were acclimatized for one week before the experiment. The rats, weighing 170g ± 10g, were housed in cages (Techniplast, Milan, Italy) with gold flake bedding (SPPS, Frasne, France) and environmental enrichment, with free access to standard chow and water. Daily, the rats were weighed and monitored for wellbeing. The sample size of 20 rats per group was calculated with *α*=5%, power of 80%, tumor development in 80% of the rats [20], and an estimated therapeutic effect of 50%.

#### 2.3.2. Tumor Implantation

The rats received tumor cells in the bladder at day 0 as described by Xiao* et al. *[21]. Before catheterization, enrofloxacin (Bayer, Leverkusen, Germany) (5-10 mg/kg) was injected subcutaneously for antibacterial prophylaxis. Experiments were performed under inhalation anesthesia: Isoflurane 2-5% (induction), followed by Isoflurane 2% and oxygen 1 L/min. The urethra was catheterized with a 16-gauge plastic intravenous cannula (BD Biosystems, Erembodegem-Aalst, Belgium) and drained. The bladder was preconditioned with a 15 sec instillation of 0.4 mL 0.1 M hydrochloride and neutralized by adding 0.4 mL 0.1 M potassium hydroxide for 15 sec. The bladder was drained and flushed 3 times with 0.8 mL 0.01 M PBS. AY27 cells were harvested, counted, and resuspended in medium to reach a concentration of 3*∗*10^6^ cells/mL. Within 30 minutes after harvesting, 0.5mL of the cell suspension was instilled in the bladder and left indwelling for 1 hour. The rats were rotated 90° every 15 minutes to optimize bladder exposure. After 1 hour, the catheter was removed.

#### 2.3.3. Study Design and Treatment

The rats were treated intravesically three, seven, and ten days after tumor cell implantation. First, the rats were anaesthetized as described before. The urethra was catheterized, and the bladder was emptied by gentle pressure on the abdomen. Group 1 was treated with 0.5mL 300*μ*mol/L curcumin-cyclodextrin complex dissolved in 0.9% NaCl. Group 2 was treated with 0.5mL BCG (BCG-Medac (Medac GmbH, Hamburg, Germany), RIVM strain 1173-P2, and 2×10^8^ – 3×10^9^ CFU). Group 3 received first an instillation with CDC for one hour and, after rinsing three times with NaCl, an instillation for one hour with 0,5 mL BCG. Group 4 received 0.5 mL 0.9% NaCl intravesically as placebo. After treatment, the bladder was emptied.

#### 2.3.4. Tumor Evaluation

At day 14, the rats were sacrificed using carbon dioxide inhalation. Internal organs were inspected for abnormalities. The bladder was removed, weighed, fixed in formalin 4% (Boom B.V., Meppel, The Netherlands), laminated in 1-2mm slices, and embedded in paraffin. Sections of 5*μ*m at two depths per lamella were stained using haematoxylin and eosin. A specialized uropathologist (C.H.K.) evaluated tumor stage according to the TNM-classification and tumor grade according to the 2004 WHO/ISUP classification. The degree of inflammation in mucosa and submucosa was quantified as no reaction, mild, moderate, or severe inflammation.

## 3. Results

### 3.1. In Vitro

**Figure 1:**
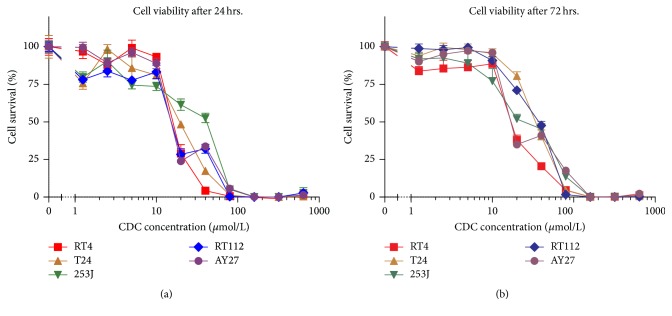
Relative cell survival of four human urothelial carcinoma cell lines and one rat urothelial carcinoma cell line (AY27) after 1 hour of treatment with cyclodextrin-curcumin (CDC). Cell viability is relative to untreated cells. The MTT cell survival assay was performed 24 hours (a) and 72 hours (b) after treatment. Error bars represent standard error of the mean (SEM). Tests were performed in quadruplicate.

One hour of CDC treatment of UC cell lines resulted in a dose-dependent antiproliferative effect (Figure 1). The 50% inhibitory concentration (IC-50) of CDC in *μ*mol/L after one and three days of incubation was 15.9 and 17.6 for RT4, 41.3 and 23.5 for 253J, 15.1 and 28.9 for AY-27, 19.3 and 34.6 for T24, and 15.2 and 37.2 for RT112. Treatment with 160*μ*mol/L CDC for 1 hour resulted in complete cell kill.

**Figure 2:**
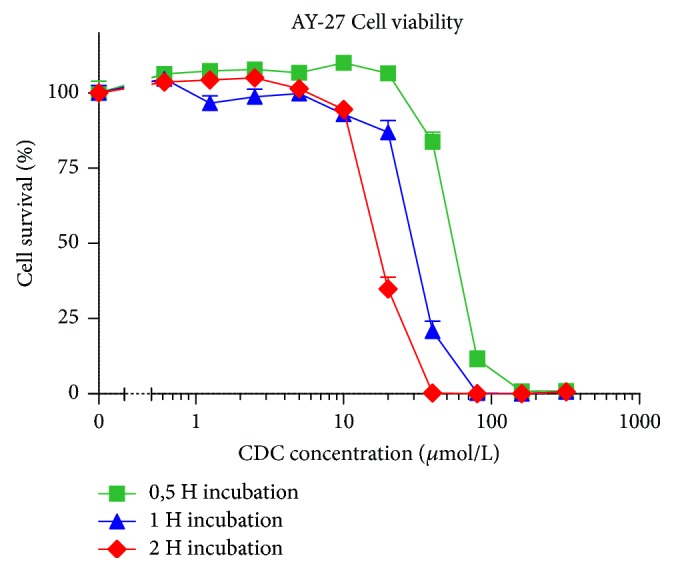
Relative cell survival of rat urothelial carcinoma cells (AY27) after treatment with cyclodextrin-curcumin (CDC) for 30, 60, and 120 minutes. Cell viability relative to untreated cells. The MTT cell survival assay was performed 72 hours after treatment. Error bars represent standard error of the mean (SEM).

The antiproliferative effect was enhanced when treatment was prolonged: when AY-27 cells were treated for 30, 60, and 120 minutes, the IC-50 of CDC decreased: 16.5 *μ*mol/L after 120 min treatment, 28.9 *μ*mol/L after 60 minutes, and 55.1 *μ*mol/L after 30 minutes (Figure 2).

### 3.2. In Vivo

#### 3.2.1. Macroscopy

The rats received three instillations with CDC, BCG, CDC+BCG, or NaCl. Two rats died during the experiment. One rat (CDC+BCG group) died one day after tumor cell instillation and was excluded from the analysis. The other rat (NaCl group) died just after the second instillation during anesthetic emergence. This rat had a pTa tumor and was included for the pathologic stage evaluation. At necropsy, no abnormalities were found on abdominal organs in both rats and the bladders were intact.

The other 78 rats recovered well from four times anesthesia and three instillations. There were no signs of impaired wellbeing or infections. Two (tumor-positive) rats (CDC+BCG and NaCl group) had hematuria but showed no other signs of tumor related problems. At necropsy, no abdominal abnormalities were found.

#### 3.2.2. Pathology

The number of tumor-free rats was eight (40%) in the group treated with CDC; five (25%) in the group treated with BCG; six (36.6%) in the group treated with CDC+BCG; and three (15%) for the NaCl group (Table 1). The number of tumor-free rats was higher in the CDC and CDC+BCG group compared to the NaCl-control, but this did not reach statistical significance (Fischer's Exact Test p=0.078 and 0.199, respectively). All tumors represented high-grade UC. Tumor stages included pTa, pT1, pT2, and pT3 and were not related to the treatment group (Kruskal Wallis test, Chi^2^=3.95, p=0.267). Fourteen days after tumor induction, the tumor progressed to a muscle invasive bladder tumor in 50.6% of the rats. Half of the pT3 tumors were seen in the NaCl group. Three of the four pT3 tumors in the NaCl group showed macroscopically extravesical extension (pT3b). Extravesical extension was not observed in the treatment groups.

#### 3.2.3. Inflammation

Nineteen rats (23.8%) showed no signs of inflammation, 46 (57.5%) showed mild, and 15 (18.8%) showed moderate inflammation of the bladder. Severe inflammation was absent. Bladder inflammation was characterized by a granulomatous reaction with influx of macrophages and polymorphonuclear lymphocytes. There was no difference in the nature or degree of inflammation between treatment and/or NaCl group(s).

## 4. Discussion

Curcumin, a food spice, is a nontoxic agent with antiproliferative activity against urothelial carcinoma (UC)* in vitro *[5, 22, 23] and* in vivo *[7–10]. Because the bioavailability of curcumin is hindered by its poor water solubility, cyclodextrin-curcumin (CDC) complex was produced to improve its solubility, stability, and bioavailability.

In this study, we show that CDC exerts a dose-dependent antiproliferative effect on human and rat UC cell lines. Moreover, intravesical treatment of CDC with and without BCG in rats bearing bladder cancer resulted in a lower number of tumor-positive rats compared to the NaCl-control group although this did not reach statistical significance.

### 4.1. In Vitro

Five UC cell lines were treated with CDC for 1 hour, to mimic the clinical situation of intravesical instillation. The IC-50 varied and ranged from 15.1 to 41.3 *μ*mol/L, likely reflecting the different phenotypes of the cell lines. The IC-50 are within the range of other* in vitro* studies on curcumin treatment of urothelial cells (3.9-20.5 uM [5]) but also other malignant cell types (10.5-14.5 uM in mammary, prostate, and ovarian carcinoma [24]). Longer exposure to CDC resulted in more cell kill of AY-27; when treatment times doubled, the IC-50 values decreased with 50%. This may have clinical implications because intravesical treatment typically lasts one hour, but extended treatment times depend on the patient's ability to retain the instillation longer.

The effects of CDC on a two-dimensional cell culture cannot directly be compared to a three-dimensional model. Differences in architecture and extracellular matrix alter the efficacy of CDC [5]. Therefore, we conducted an* in vivo* experiment.

### 4.2. In Vivo

To resemble the clinical situation as much as possible with consecutive intravesical instillations, we used the orthotopic bladder cancer model in F344 rats with syngeneic AY-27 UC cells. Rats were treated with an intravesical instillation three, seven, and ten days after tumor cell implantation and sacrificed on day 14. BCG and a combination of CDC+BCG were included as treatment modalities since BCG is standard treatment for NMIBC [2] and curcumin may potentiate the efficacy of BCG [7].

We demonstrate that treatment with CDC and CDC+BCG results in a lower percentage of tumors (60% and 68%, respectively) compared to NaCl-control (85%). This difference was not statistically significant (p=0.078 and 0.199, respectively), but we observed that the majority of high-stage tumors were present in the NaCl and BCG-treated rats. In this aggressive model, it is encouraging that we observed less and lower-staged tumors in the CDC-treated groups.

Curcumin is under investigation as possible therapeutic moiety for a variety of malignancies, including urological tumors. Only few* in vivo* studies report intravesical treatment with curcumin or CDC for a urological malignancy [8–10], but not in combination with BCG. Leite* et al.* used an orthotopic mouse model with MB49 UC, treated twice weekly with an intravesical instillation of curcumin [8]. They report a significant reduction in tumor size for the treated mice, compared to placebo, but no effect on number of tumors or invasion depth. Another* in vivo* study, using the same orthotopic bladder cancer mouse model, treated animals with four intravesical treatments of curcumin [10]. They report a positive effect on tumor necrosis in the curcumin treatment rats, but no effect on tumor size or number. Opposed to these studies, our experiment used tumor-free rats as the endpoint. By using the cyclodextrin-curcumin complex, we aimed at higher cellular uptake and improved efficacy because of improved bioavailability [17, 19, 25] compared to unmodified curcumin used in these studies.

Curcumin and BCG share pathways responsible for a tumor selective antiproliferative effect by suppressing transcription factors, like NF-*κ*B, responsible for proliferation, invasion, and angiogenesis [7, 11–13], and by inducing apoptosis via TRAIL [7, 15].* In vitro* and* in vivo* evidence indicate additive effects of curcumin and BCG treatment for UC [7]: Intratumorally administered BCG combined with oral fed curcumin immediately after tumor grafting resulted in a significant reduction in tumor volume compared to BCG alone, curcumin alone, or placebo. Although this model is a poor reflection of the clinical situation, it shows the possible potentiating effect of curcumin [7]. Comparing this study to our results is difficult, since the bioavailability of both curcumin and BCG differs greatly after oral and intratumoral administration, compared to intravesical instillation respectively. Moreover, in our treatment schedule animals were treated after tumor establishment, i.e., in a therapy setting, whereas the other study was more of a protective setting. Our experiment did not show an additional or synergistic effect of the combination CDC+BCG over CDC alone (31.6% tumor-free rats versus 40%, respectively). The number of tumor-positive rats per group was too low to observe statistically significant results between these groups. But interestingly, for tumor-positive rats, the combination treatment resulted in a slightly higher ratio of NMIBC/MIBC compared to the CDC treatment: 6/7 versus 4/8. This indicates a positive treatment effect compared to BCG alone (2/13) in the spectrum of tumor development from NMIBC to MIBC.

Combination treatments of an immunomodulator with a chemotherapeutic (like Mitomycin-C, Gemcitabine, Epirubicin, etc.) or with another immunomodulator (Curcumin, Interferon) have shown favorable results [7, 26]. Advantages of combining agents may lie in a potentiating effect on uptake or synergistic stimulation of apoptosis factors, as described before. Future research will focus on the combining well-known agents with new or established treatments.

The results of BCG treatment in our experiment are mediocre. Xiao reports a lower percentage of tumor-bearing rats (58%) after BCG treatment in the same AY27 model, compared to us (75%) [27]. Two other* in vivo* experiments show a comparable and more modest result of BCG with 64% and 75% tumor-positive mice after intravesical BCG treatment [28, 29]. BCG treatment showed a limited antitumor effect compared to clinical data. Possibly, the evaluation time was too short as the induction of the effector T-cells responsible for the antitumor effect requires a number of days. Moreover, the magnitude of T-cell response increases synergistically with each treatment and may need weeks to fully exert its effect [30]. Longer follow-up after treatment may be preferable, but because of the aggressiveness of the model, we chose to sacrifice the animals after two weeks. Xiao* et al*. used a different study design; they monitored F344 rats with AY-27 tumors for 90 days and euthanized animals when clinical signs of tumor progression became apparent [27]. Survival studies might have shown differences between treatment groups, but considering the advanced tumors in many animals, this seems unlikely for our experiments.

Our results indicate that the model used may be too aggressive to evaluate investigational agents that need a certain amount of time to fully exert their antitumor effect. The AY-27 orthotopic bladder cancer rat model used was first described by Xiao* et al*. Muscle invasive tumors are to be expected, since the urothelium first needs to be damaged before tumor cells can attach and grow [21]. The model appears to be more aggressive than originally described. Hendicksen* et al.* assessed the tumor growth over time in the AY27 model and found that, after 6 days, already 40% of the rats progressed to MIBC (pT2, pT3) [20]. Considering that both curcumin and BCG work as immunomodulators, rapid tumor development may overshadow the antitumor effect.

Due to limited penetration depth, intracellular concentration of the drug is lower at the base of larger tumors, contributing to possible lower treatment effects. In future* in vivo* studies, ideally the tumor load before start of the treatment should be limited to assure tumor cell-drug contact.

## 5. Conclusions

Being a nontoxic agent, curcumin possesses a variety of properties making it a potential cancer treatment or enhancer of existing treatments. The cyclodextrin-complexed formulated curcumin with improved bioavailability showed a dose-dependent antiproliferative effect on the rat AY-27 and various human UC cell lines* in vitro*. Intravesical instillation of CDC for the treatment of bladder cancer with and without BCG demonstrated a promising antitumor response. Extrapolating to the clinical situation, (adjuvant) treatment with CDC deserves a successive study.

## Figures and Tables

**Table 1:** Pathological stage per treatment group. CDC: cyclodextrin-curcumin, BCG: Bacillus Calmette Guérin, NMIBC: non-muscle invasive bladder cancer, and MIBC: muscle invasive bladder cancer.

Treatment group	Tumor free pT0 N (%)	NMIBC pTa, pT1 N (%)	MIBC pT2, pT3 N (%)	Total N (%)
(1) CDC	8 (40.0)	4 (20.0)	8 (40.0)	20 (100)

(2) BCG	5 (25.0)	2 (10.0)	13 (65.0)	20 (100)

(3) CDC+BCG	6 (31.6)	6 (31.6)	7 (36.8)	19 (100)

(4) NaCl (control)	3 (15.0)	5 (25.0)	12 (60.0)	20 (100)

Total	22 (27.8)	17 (21.5)	40 (50.6)	79 (100)

## Data Availability

The* in vitro* and* in vivo* data that support the findings of this study are included within the article. Requests for access to additional (raw) data will be considered by the corresponding author.
